# News and Events of the Week

**Published:** 1911-04-29

**Authors:** 


### News and Events of the Week.

Dr. M. S. Pembrey will lecture on "The Physiological Principles of Physical Training" on Wednesday, May 3, at 5 p.m., at the Royal Sanitary Institute, 90 Buckingham Palace Road, S.W.

The Ingleby Lectures of the University of Birmingham will be given on Thursdays, May 18 and 25, at 4 o'clock, by Dr. E. F. Bashford. Subject: Advances in Knowledge of Cancer.

Professor Richet, member of the Paris Academy of Medicine, has founded a prize at the "Société des gens de lettres," for the purpose of recompensing "a doctor who has carried out a work of imagination." The reward is to be granted every second year, and is of the value of twenty pounds.

We have received a supplement to the catalogue of Lewis's Medical and Scientific Circulating Library. The original catalogue only dealt with works published up to the end of 1907. The present supplement includes a classified index of subjects and authors from 1908 to the end of 1910. It is published at 6d., but will be presented gratis to subscribers to the Library on application to H. K. Lewis, 136 Gower Street, London, W.C.

The mortality from diabetes in Paris has shown an extraordinary increase of late years. In 1880 only 128 such deaths were registered, in 1890 the number was 304, in 1900 it had risen to 427, in 1908 to 464, and a year later to 525. Thus the number of deaths has quadrupled in thirty years. This disquieting increase results from the spread of the disease, and also from the fact that most of its victims refuse to follow proper treatment, or at any rate to carry it out thoroughly.

A recent number of this journal contains a translation of a letter written by a member of the French Consular Service with reference to the death of Dr. Mesny, one of the victims of duty in the recent outbreak of plague in Manchuria. A committee has just been formed in Paris to draw up plans for the erection of a memorial to this medical martyr. This memorial is to be erected at Brest, where Dr. Mesny was born in 1869. It is hoped, however, that the memorial will receive the support of all Frenchmen, and to that end the presidency of the committee will be offered to the French Minister of War. Subscriptions may be sent to M. Armand Guillon, 9 due de Poissy, Paris, Ve.

A ball will be held at the Empress Rooms, Royal Palace Hotel, Kensington, W., on Monday, May 15, 1911, in aid of the funds of the Women's Sick and Wounded Convoy Corps, under the patronage of Her Highness, Princess Marie Louise of Schleswig-Holstein. This organisation trains and forms from amongst its members women's voluntary aid detachments, which are registered at the War Office through the British Red Cross Society for employment as technical reserves in time of national danger and emergency. Tickets and any further particulars may be obtained from the Committee, and Capt. Langford Lloyd, D.S.O., R.A.M.C., Hon. Secretary Ball Committee, or the Secretary Women's Sick and Wounded Convoy Corps, 39 Great Smith Street, Westminster, S.W.

A new French society has been formed with the object of providing open spaces and playgrounds for the children of the working-classes and to encourage every form of open-air exercise.

The results of the census taken on December 31, 1910, simultaneously in Austria, Hungary, and Bosnia-Herzegovina are to hand. The total population amounts to 51,314,271, of which Austria claims 28,567,898 (an increase of 9.2 per cent. in ten years), Hungary 20,850,700 (an increase of 8.3 per cent. in ten years), and Bosnia-Herzegovina 1,895,673 (an increase of 30.89 in five years).

Probably few people realise that, with practically only one exception, suicide is responsible for more deaths annually in Paris than any other disease. Putting on one side tuberculosis in all its forms, which is responsible for a quarter of the deaths, or 12,000 out of 45,000 deaths notified, and accidental deaths by violence, which numbered 941, the only disease which claimed more victims than suicide was pneumonia, which killed 1,402 people.

Mr. Havelock Ellis has written a critical and biographical introduction to an English translation of "Love and Marriage," by Ellen Key, the well-known Swedish author of "The Century of the Child." The treatise is to be published immediately by Putnams. Whatever may be thought of Ellen Key's opinions, as Mr. Havelock Ellis says, "Her writings are the candid expression of her intimate self." The book is published in six languages.

We have received a copy of the Dentists' Register for 1911, from which we gather that 4,883 dentists are registered in the United Kingdom, of whom 3,124 (or 63.97 per cent.) are graduates or licentiates of various Universities and colleges, 11 in bona-fide practice of dentistry holding additional surgical qualifications, and 1,725 in bona-fide practice without additional qualifications. Four Colonial dentists are registered in England, and 19 dentists with qualifications obtained from the American Universities of Harvard or Michigan.

According to a report of the Italian Minister of Agriculture with regard to the commission of experts appointed to search for springs of drinking water in the neighbourhood of the Bari district, the commissioners were obliged, after making use of "water-finders," to sign a declaration before a notary in the presence of the municipal authorities that certain borings made as the result of the "findings" of these worthy people had not yielded the looked for supplies of fresh-water. Nothing less than this procedure would convince the people of the powerlessness of this occult science. If one does not at sight deny the success of the divining rod, it would be interesting to know the part that electricity plays in the matter. It intervenes no doubt much in the same way as magnetism did a few years ago in the practice of successful hypnotisers.

An Imperial Health Fête will be opened by H.R.H. Princess Louise Duchess of Argyll, at the Royal Botanic Gardens, on Wednesday, July 5. It has been organised by the Women's Imperial Health Association (which has conducted so vigorous and successful a Caravan Health Crusade amongst English villages), and expects to be one of the most notable society functions in this memorable year. The Fête will precede a two-days Congress on health subjects, and is designed to draw the attention of the British Dominions beyond the seas to the Association's practical propagation of health topics amongst the masses. A marquee will be devoted to each of the British Colonies, in which typical women and girls' home and club industries will be shown. The "Florence Nightingale" Caravan of the Association, which has done such good work amongst the villages in the Eastern Counties, will be on view equipped as on tour; and demonstrations including the display of "Health" cinematograph films will be given in the Gardens.

The Annual General Meeting of the Medical Defence Union will be held at the Grand Hotel, Brighton, on Thursday, May 18, at 4.30 p.m., when the annual report of the Council for 1910 will be duly presented to members.

Dr. Yamei Kin, who is visiting England on a tour of hospital inspection, was one of the speakers at the Anti-Opium Congress on Monday. Dr. Kin studied and obtained her medical degree—the first granted to a Chinese lady—at New York, and is in charge of the Government Hospital near Tsien-Tin. She is keenly interested in the movement to train native Chinawomen as nurses, and has done pioneering work in this direction.

The following appointments and reappointments have been made at the Manchester Royal Infirmary: Re-appointments: Mr. Cecil Hibbert, M.B., Ch.B. Vict., and Mr. J. W. Bride, M.B., B.S. Lond., Medical Officers at the Central Branch; Mr. George Ashton, M.D., Vict., Medical Officer for Home Patients. Appointments: Mr. E. R. Eatock, M.B., Ch.B. Vict., and Mr. J. F. Cocker, M.B., Ch.B. Vict., Junior House Surgeons; Mr. E. Gray, M.B., Ch.B. Vict., House Physician.

